# Analgesic efficacy of ultrasound guided paravertebral block in percutaneous nephrolithotomy patients: a randomized controlled clinical study

**DOI:** 10.1186/s12871-020-01169-6

**Published:** 2020-09-29

**Authors:** Ferda Yaman, Devrim Tuglu

**Affiliations:** 1 Department of Anesthesiology and Reanimation, Faculty of Medicine, University of Eskişehir Osmangazi, 26040 Eskişehir, Turkey; 2 Department of Urology, Faculty of Medicine, University of Kırıkkale, Kırıkkale, Turkey

**Keywords:** Ultrasound, Paravertebral block, Percutaneous nephrolithotomy

## Abstract

**Background:**

Paravertabral blocks (PVB) are in use to adequately manage pain arising from a variety of operations on the thorax, abdomen or pelvis. PVB is straightforward, efficacious in operations performed. This study was undertaken to evaluate how efficacious ultrasound-guided thoracic paravertebral block is when used in patients undergoing percutaneous nephrolithotomy (PCN).

**Methods:**

A total of 44 patients, falling in categories I to III of the American Society of Anesthesiologists, and aged between 18 and 65 years, who were scheduled for PCN, were randomly distributed into two groups. The anaesthetic intervention group (PVB) contained 22 individuals, who were injected at level T8-T9 with 20 mL 0.25% bupivacaine as a single administration. In the control group C, also containing 22 individuals, the intervention was not carried out. The groups were compared after PCN in terms of opioid use, pain score, opioid adverse effects profile and the need for supplemental analgesia.

**Results:**

Visual analogue scale pain scores whilst at rest or moving were lower at the level of statistical significance in the PVB group compared to controls at 2 and 4 h post-surgery. At 6 and 8 h post-surgery, the control group had a lower VAS score when moving, and this result reached statistical significance (*p* < 0.05). The controls used more opioid relief than the PVB group and had lower scores for satisfaction (*p* < 0.05).

**Conclusion:**

Ultrasound-guided PVB using bupivacaine and an in-plane technique provides effective analgesia in PNL. It is associated with high scores on patient satisfaction and minimal complications.

**Trial registration:**

ClinicalTrials.gov, NCT04406012. Registered retrospectively, on 27 May 2020.

## Background

PCN (percutaneous nephrolithotomy) is a frequently employed, minimally invasive operative technique technique used to remove renal calculi [1]. The technique remains associated with significant demand for analgesic interventions post-surgically. The application of regional anaesthesia is known to possess the highest efficacy in managing pain following surgery of this sort [2]. There are a number of methods available which may potentially reduce postoperative pain associated with nephrostomy tube placement in PCN, namely intercostal nervous blockade, epidural analgesia, peritubal infiltration of local anaesthetic and paravertebral blockade [3–5]. Paravertabral blocks (PVB) are in use to adequately manage pain arising from a variety of operations on the thorax, abdomen or pelvis. PVB is straightforward, low risk and is efficacious in operations performed unilaterally. It rarely creates hypotension, urinary retention or nausea and vomiting following surgery [6]. PVB performed under ultrasonic guidance (PVB-US) targets the region of emergence of the spinal nerves through the foramina of the vertebrae. It blocks somatic and sympathetic fibres supplying several adjacent dermatomal segments both superior and inferior to where the injection is given [7].

The study’s main aim was evaluating analgesic efficacy in PVB-US to the thorax, whilst the secondary aim was assessment of how satisfied patients were with the procedure and how much rescue analgesia was needed in such cases.

## Methods

**Fig. 1:**
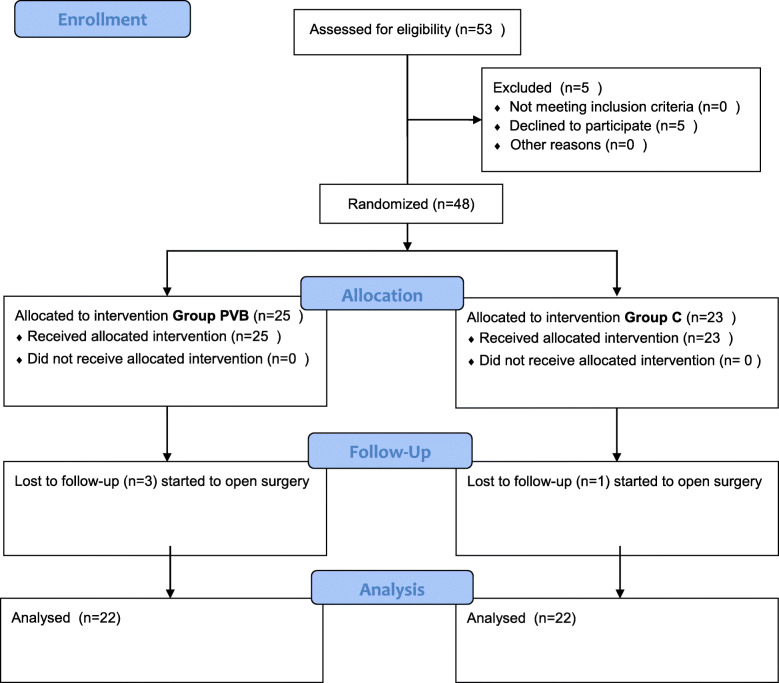
CONSORT flow diagram. Consolidated Standards of Reporting Trials (CONSORT) flow diagram showing patients’ recruitment and allocation

Ethical approval was obtained from the Local Ethics Committee of Kırıkkale University, Kırıkkale, Turkey (No.04/03) and registered retrospectively on the ClinicalTrials.gov database under registration number NCT04406012. The inclusion criteria of this study were an age of 18–65 years and an American Society of Anesthesiologists classification of I or III scheduled for PCN from February 2016 to july 2016. Patients participating the study is shown in the CONSORT diagram (Fig. 1). (No.04/03) and registered retrospectively on the ClinicalTrials.gov database under registration number NCT04406012. Of the 53 individuals with eligibility to join the trial, 5 refused to join and 4 had significant haemorrhage during PCN, so that open surgery was then needed. Thus 44 individuals, ranging from category I to III in the classification scheme of the American Society of Anesthesiologists, were actually enrolled. All trial participants provided written, informed consent. The exclusion criteria were: age below 18 years; current pregnancy; allergy to local anaesthetic drugs; bleeding disorder; depressive illness or anxiety disorder; being obese (i.e. BMI above 35); previous pneumothorax; phrenic nerve paralysis; stenotic aorta of severe degree. The participants in the trial were allocated into one of two groups – those receiving the anaesthetic intervention (thoracic paravertebral block: PVB) and control subjects – using the closed envelope randomisation technique 1 h prior to surgery before admission to the operating room by the nurse of urology yard. Monitoring electrocardiogram (ECG), peripheral pulse oximetry and external blood pressure measurement) was set up at 5 min intervals.

Postoperative analgesia requirements were evaluated using the visual analogue scale (VAS), which ranges in value from 0, indicating an absence of pain, to 10, indicating pain of high severity. The evaluations were performed at 1, 2, 4, 6, 8, 10, 12 and 24 h post-surgery. The score was checked when the patient was resting and when moving (such as when deeply inspiring or coughing). A VAS score above 4 was taken to indicate a need for extra pain relief. The control group were given a standard post-surgical analgesia regime, i.e. dexketoprofen 50 mg i.v. Where administering dexketoprofen did not result in a VAS score below 4, tramadol was co-administered at a dosage of 1 mg per kg body weight.

The individuals in the PVB group all received paravertebral blocks. This group all underwent monitoring via ECG, external BP measurement and pulse oximetry within theatre before PVB was undertaken. Anaesthetic induction occurred after PVB placement. The patient assumed a seated position and a linear 10–18 MHz ultrasound probe (EsaoteMyLab 30, Geneva, Italy) was positioned paramedially over the space between two transverse processes. The transverse processes of the T9 and T10 vertebrae and the superior pleura and costotransverse ligament were visualised. An 80 mm long needle of 22 gauge (Pajunk, Geisingen, Germany) was inserted under ultrasonic guidance and 10 mL bupivacaine hydrochloride (Marcaine 0.5%, Astra Zeneca) dissolved in 20 ml solution was infiltrated into the paravertebral space. The needle is advanced to the point where the superior costotransverse ligament crosses the space. The dispersal of the local anaesthetic agent over the pleura was observed, along with its displacement. All PVBs were undertaken by the same, experienced anaesthetist.

After the paravertebral block was performed, all patients received standardized general anesthetic technique with 2–2.5 mg kg^− 1^ propofol, 0.6 mg kg^− 1^ rocuronium, 1–2 mcg kg^− 1^ fentanyl and anesthesia was maintained with sevoflurane and oxygen-air mixture. Intraoperative dose of additional opioid was different and noted in anesthesia follow-up form.

The pain scores using VAS were noted in both groups post-procedure. The degree of patient comfort was also noted. The occurrence of nausea and vomiting and any need for further pain relief was noted. At 24 h post surgery patient satisfaction was noted. A scale of 1 to 5 was employed for this, ranging from 1 (very unsatisfied) to 5 (very satisfied). The same urologist performed the surgery in all cases.

A power calculation was performed with the G * Power 3.1.9.4 statistical package application using the following parameters: n1 = 22, n2 = 22, α = 0.05, (effect size) d = 0.9; (power (1-β)) = 0.83. The data obtained were evaluated using the IBM SPSS 25.0 statistical application. The Chi-Square statistic was used in comparisons. Descriptive statistics were obtained for the data (frequency, percentage, mean, standard deviation, median, min-max), both continuous and categorical. The data were tested for normal distribution using the Shapiro-Wilk test. The independent samples t Test was employed for comparisons involving normally distributed quantitative data in the groups, and the Mann–Whitney U test was used in the analysis of non-normally distributed data. The paired samples t test (t test in dependent groups) was employed for within-group comparisons. A *p* value below 0.05 was taken to indicate statistical significance.

## Results

The groups did not differ at the level of statistical significance in terms of sex, age, weight, BMI or classification under the ASA rubric (*p* > 0.05). Both groups were the same from a statistical point of view in terms of opioid use during the surgery and in post-surgical satisfaction score (p > 0.05). In the post-surgical period, group C used a greater amount of opioid for pain relief than group PVB, and they were less satisfied with the procedure overall (*p* < 0.05). See Table 1.

**Table 1:** Demographic data of patients, intraoperatively opioid consumption and patient satisfaction scores between groups

		Block (*n* = 22)	Control (*n* = 22)	P
Gender	female	6 (%27,3)	12 (%54,5)	0,125 ^a^
	male	16 (%72,7)	10 (%45,5)	
Age (year-old)		50,3 ± 10,5	48,7 ± 14,1	0,673 ^b^
Body Mass Index (kg/m^2^)		28,0 ± 4,1	30,7 ± 5,2	0,059 ^b^
ASA	I	5 (%22,7)	2 (%9,1)	0,447 ^a^
	II	15 (%68,2)	17 (%77,3)	
	III	2 (%9,1)	3 (%13,6)	
Intraoperative opioid consumption		98,0 ± 27,5	132,3 ± 39,8	**0,002** ^b^
Patient Satisfaction Score		4,0 ± 0,7	3,0 ± 0,7	**0,000** ^b^

^a^Chi Square Test

^b^Independent Samples t Test

**Fig. 2:**
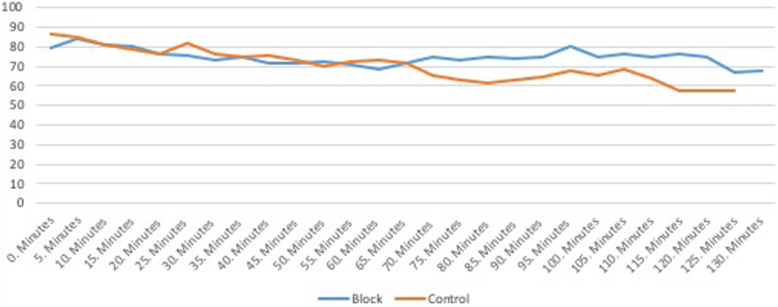
Changes in Mean Arterial Pressure between the groups intraoperatively

**Fig. 3:**
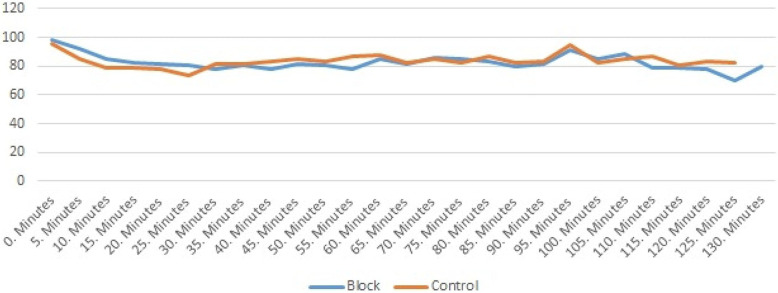
Changes in Heart Rate between the groups intraoperatively

The length of time for the operation was between 115 and 127 min. Figure 2 provides the variation in mean arterial pressure and Fig. 3 the cardiac rate during surgery.

At 1 h post-operatively, the VAS score was higher during movement (deep breath, “dynamic”) than at rest and this differnce attained statistical significance. In group C, VAS was higher during movement than at rest (*p* < 0.05).

At 2 and 4 h post-operatively, the VAS score was higher in group C, both at rest and while moving. These results were statistically significant.

At 6 and 8 h post-operatively, the VAS “at rest” score did not differ significantly between groups (*p* > 0.05) but the VAS score “moving” was significantly higher in group C (p < 0.05). Additionally, the VAS scores differed significantly within the C group when “at rest” and “moving” scores were compared (*p* < 0.05), with the latter being higher, i.e. indicating more discomfort.

At 10 and 12 h post-operatively, the VAS “moving” scores were significantly higher in group C (*p* < 0.05).

At 24 h post-operatively, both dynamic and resting VAS scores did not differ at the level of statistical significance, neither resting nor moving (*p* > 0.05). See Table 2: Evaluation of VAS scores at rest and while moving (mean ± SD)).

**Table 2:** Evaluation of the visual analog score at rest (Mean ± SD)

VAS		Block (*n* = 22)	Control (*n* = 22)	P*
1. hour	rest	0,5 ± 1,2	2,5 ± 3,2	**0,016**
	dynamic	0,7 ± 1,5	2,8 ± 3,5	**0,014**
	P**	0,083	**0,017**	
2. hour	rest	2,4 ± 2,1	4,6 ± 3,5	**0,014**
	dynamic	2,8 ± 2,2	5,0 ± 3,9	**0,022**
	P**	**0,001**	**0,025**	
4. hour	rest	1,5 ± 1,2	3,2 ± 3,2	**0,029**
	dynamic	1,9 ± 1,5	4,0 ± 3,5	**0,011**
	P**	**0,002**	**0,000**	
6. hour	rest	1,3 ± 1,1	3,0 ± 2,6	**0,010**
	dynamic	1,5 ± 1,4	4,0 ± 2,9	**0,001**
	P**	0,104	**0**	
8. hour	rest	1,2 ± 1,2	2,2 ± 2,2	0,071
	dynamic	1,4 ± 1,3	3,0 ± 2,6	**0,016**
	P**	0,057	**0,000**	
10. hour	rest	1,0 ± 1,0	1,8 ± 2,0	0,079
	dynamic	1,3 ± 1,2	2,1 ± 2,5	0,172
	P**	**0,005**	0,056	
12. hour	rest	0,6 ± 0,8	1,0 ± 1,6	0,354
	dynamic	0,9 ± 1,2	1,3 ± 2,0	0,462
	P**	**0,011**	0,056	
24. hour	rest	0,2 ± 0,7	0,4 ± 0,8	0,413
	dynamic	0,0 ± 0,2	0,5 ± 1,3	0,080
	P**	0,186	0,162	

* Independent Samples t Test

** Paired Samples t Test

There was a statistical difference found in the rate of requiring extra pain relief at 1, 2, 4, 8 10, 12 and 24 h post-surgically. In all cases, group C had greater need for analgesia (*p* < 0.05). See Table 3.

**Table 3:** Additional analgesic consumption between the groups

Additional tramadol		Block (*n* = 22)	Control (*n* = 22)	P^a^
1. hour	no	22 (%100,0)	20 (%90,9)	0,488
	yes	0 (%0,0)	2 (%9,1)	
2. hour	no	13 (%59,1)	9 (%40,9)	0,366
	yes	9 (%40,9)	13 (%59,1)	
4. hour	no	21 (%95,5)	18 (%81,8)	0,345
	yes	1 (%4,5)	4 (%18,2)	
6. hour	no	21 (%95,5)	14 (%63,6)	**0,021**
	yes	1 (%4,5)	8 (%36,4)	
8. hour	no	22 (%100,0)	20 (%90,9)	0,488
	yes	0 (%0,0)	2 (%9,1)	
10. hour	no	22 (%100,0)	19 (%86,4)	0,233
	yes	0 (%0,0)	3 (%13,6)	
12. hour	no	22 (%100,0)	22 (%100,0)	1000
	yes	0 (%0,0)	0 (%0,0)	
24. hour	no	22 (%100,0)	22 (%100,0)	1000
	yes	0 (%0,0)	0 (%0,0)	

^a^Chi Square Test

The two groups did not differ at the level of statistical significance for nausea and vomiting at any point (*p* > 0.05).

No complications, e.g. local anaesthetic toxicity, vascular puncture, pneumothorax, inadvertent epidural injection or spinal anesthesia were noted.

## Discussion

The satisfaction score following percutaneous nephrolithotomy was high in the group PVB, indicating that PVB is sufficient to provide post-surgical analgesia after such a procedure. Up to 6 h after surgery, the individuals who underwent PVB scored significantly better on VAS than those in whom this anaesthetic procedure was not undertaken. This superiority did not persist after the 6 h post-surgical interval.

The results from this study are in line with other published research on using PVB in PCN. However, there are disparities in the other studies in terms of timing, optimal concentration of local anaesthetic and the level at which to inject. It is also unclear whether one or more infiltrations of local anaesthetic are needed [5, 8–11].

Different techniques have been employed previously for PVB. A technique relying on loss of resistance has been described, which was in use before ultrasound guidance became available within operating theatres. Ak, K. et al. described injecting 0.5% levobupivacaine at the level of the tenth, eleventh and twelfth thoracic vertebrae. 4 mL were infiltrated at each level as the surgery, conducted under general anaesthesia, ended. These researchers noted that pain at rest as assessed by VAS was significantly improved following PVB, for 2 h post-surgery [10]. The present study differed in how PVB was used. In the present study, the patient was conscious and sitting upright as 20 mL bupivacaine 0.25% was infiltrated. We found pain relief lasted for 6 h post-surgically. The difference in observed outcome compared to other studies may relate to the difference in injection volume and the prolonged action duration of bupivacaine compared to levobupivacaine.

Hatipoğlu et al. reported on performing PVB at levels T11, T12, L1 prior to surgery but with the patient under general anaesthesia and placed in a prone position. They employed 0.5% bupivacaine, injecting 5 ml per level under ultrasound guidance. Thus the total volume of local anaesthetic was 15 mL. These authors state that analgesia was mantained up to 24 h post-surgery [9]. Our findings reveal that the VAS-rated discomfort was significantly lower in group PVB than in group C until 12 h post-surgery when the patient was moving, but not while at rest. We performed the procedure without patient sedation, with the patient sitting upright before the operation.

Patient positioning may affect the distribution of local anaesthetic from a single injection when the concentration is low. This may be the explanation for the difference of duration of analgesia.

Yayık et al. investigated analgesia procured through PVB vs peritubal infiltration. VAS dynamic and resting scores were significantly lower in the PVB group than the peritubal infiltration group or a control group at all time points following surgery up to 24 h post-surgery. They performed the PVB procedure with the patient in the prone position. They employed 0.25% bupivacaine injected at levels T8–9, at the end of surgery [11]. Our study used the same concentration and volume of bupivacaine, however PVB was undertaken prior to surgery.

A different study also employed PVB in percutaneous nephrolithotomy. A catheter was inserted into the paravertebral space at level T10 prior to commencing surgery. Catheter insertion was in awake patients, sitting upright. Ultrasonic guidance was not used. Twenty mL bupivacaine 0.5% was injected prior to surgery. Rescue analgesia was noted to be required first at 275 min post-surgery [8]. The duration of analgesia achieved with this volume and concentration indicates that PVB involving a single injection is insufficient for complete analgesia postoperatively over the first 24 h post-surgery. However, it does lead to a decreased need for systemic analgesic drugs. In our study, we used a lower concentration but the same volume of bupivacaine. For the first 4 h post-surgery, VAS scores, both dynamic and resting, were significantly lower in group PVB than in the control group. Our results indicate that the duration of PVB is 6 h using bupivacaine 0.25% in 20 mL total volume.

Baldea et al. report on a study in which PVB block was performed at the level of T10 by means of a single injection of 20 mL 0.5% bupivacaine. The block was performed prior to surgery with the patient seated and under ultrasound guidance. The first dose of opioids for relief analgesia was given at 119.7 min post-surgically in the PVB group [12].

It is clear that PVB is efficacious in providing analgesia for percutaneous nephrolithotomy. Indeed, PVB, together with epidural anaesthesia, are considered Gold Standard procedures. Newer studies have focused on lengthening the duration of analgesia through the addition of adjuvant therapy, notably clonidine and dexmedetomidine [13, 14].

Kamble et al. compared PVB for PCNL using either 0.5% Bupivacaine alone or 0.5% Bupivacaine plus 1 μg/kg of clonidine: PVB was performed prior to surgery in awake patients in the sitting position. Clonidine was shown to have an adjunctive role with bupivacaine, providing a higher quality paravertebral block and prolonging analgesia to a significant extent post-surgically. The dosage employed took account of patient weight: 15 ml in patients with a weight below 60 kg and 18 ml in patients with a weight exceeding 60 kg [13]. Our study, did not address whether adjuvant pharmacological agents affect the duration of analgesia, although it did establish that a single injection of 0.25% bupivacaine in 20 mL volume produced an analgesic effect lasting 8 h after surgery ended.

Another study examined the use of PVB in video-assisted thoracic surgery (VATS). In that study, two treatments were compared: ropivacaine 0.5% in a volume of 30 mL with adjuvant dexmedetomidine 50 microgram or ropivacaine 0.5% in 30 mL alone. The level of injection was between T3 and T5. Two injections were given in the lateral decubitus position. The treatment was post-surgical but before the patient recovered consciousness. Adjuvant dexmedetomidine lengthened the duration of analgesia obtainable with bupivacaine alone. The pain score at rest (assessed using VAS) did not differ significantly at any point postoperatively, with the exception of 4 h post-surgery. In the adjuvant therapy group, the maximum VAS pain scores for the 24 h post-operative period while resting or when coughing were lower than those seen in the group receiving ropivacaine alone. This result attained statistical significance [14]. It seems that further research is needed to determine the optimal dose of local anaesthetics with or without adjuvant and to clarify the ideal timing to perform PVB, i.e. before surgery or post-surgically.

Our study showed that patient satisfaction was higher in the PVB group than group C. If PVB is performed in awake patients, a single injection may be preferable to multiple injections. Research on cadavers demonstrated that the spread of infiltrated anaesthetic was no different, whether injection occurred singly or at two levels. This study also employed ultrasonic guidance [15]. Additionally, some research has evaluated single injection vs multiple injection in PVB to the thorax. The trial participants underwent VATS, after which they had PVB using nerve stimulators to guide the injection. In terms of efficacy, the single puncture technique was potentially superior to multiple puncture, since the patients were more satisfied, the procedure took less time and there was a lower risk of developing complications [16].

Whilst PVB has become the de facto Gold Standard in chest surgery, this has yet to be acknowledged in the literature on the subject [17]. A number of studies have demonstrated the safety and efficacy of PVB to provide analgesia perioperatively in procedures affecting the kidney [18, 19].

The reports published so far about the occurrence of complications mention a risk of inadvertent epidural or intrathecal injection in approaching 1% of cases. Ultrasound was not used for guidance where this occurred. Total spinal anaesthesia has occurred on some occasions [20]. Total spinal anaesthesia has even occurred once when ultrasonic guidance was in use, but this case was approached with an out-of-plane technique [21]. This study followed a retrospective design to assess the degree of complications associated with single-puncture, transverse, in-plane PVB with ultrasonic guidance. All participants underwent mastectomy. Some 1427 PVBs were performed on the thorax, with no more than 6 complications occurring. Amongst other complications, bradycardia leading to symptoms with hypotension (*n* = 3), one vasovagal attack (*n* = 1), and a potentially toxic reaction to the local anaesthetic (*n* = 2). Neither inadvertent rupture of the pleura nor a pneumothorax leading to symptoms occurred [22].

Both in this study and in the authors’ routine practice, the authors have a preference for the in-plane anaesthetic technique. Complications of the technique, including bleeding or technical issues, did not occur.

This study suffers from certain limitations. For example, we were unable to assess precisely the tramadol dosage needed, since patient-controlled analgesia, which would give a clear picture, was not used. In addition, the area of block achieved was not precisely delineated by performing a sensory neurological examination.

## Conclusion

Ultrasound-guided PVB using bupivacaine and an in-plane technique provides effective analgesia in PNL. It is associated with high scores on patient satisfaction and minimal complications.

## Data Availability

The datasets used and/or analyzed during the current study are available from the corresponding author on reasonable request.

## Abbreviations

PVB: Paravertebral block
BP: blood pressure
VAS: Visual analogue score
PCN: Percutaneous nephrolithotomy
PVB-US: PVB performed under ultrasonic guidance
ASA: Classification of American Society of Anesthesiologists
ECG: Electrocardiogram
BMI: Body Mass Index
